# Exploring the Feasibility and Patient Acceptance of RAGT for Overground Ambulation in Adults With Guillain–Barré Syndrome: A Scoping Review

**DOI:** 10.1016/j.arrct.2026.100596

**Published:** 2026-02-01

**Authors:** Joon Sin Ser, Siew Kwaon Lui

**Affiliations:** a Rehabilitation Medicine, Singhealth Residency, Singapore; b Department of Rehabilitation Medicine, Singapore General Hospital, Singapore

**Keywords:** Ambulation, GBS, RAGT, Rehabilitation

## Abstract

**Objective:**

To review the current literature on the feasibility as well as weaknesses and strengths of robot-assisted gait training (RAGT) in the rehabilitation of gait in adult patients with Guillain–Barré syndrome (GBS).

**Data Sources:**

This scoping review was conducted in accordance with the Preferred Reporting Items for Systematic Reviews and Meta-analysis for Scoping Reviews guidelines. A systematic search between November 2024 and May 2025 of PubMed, Cochrane Library, Embase, Canadian Agency for Drugs and Technologies in Health (CADTH) Grey Matters, and citation mining was performed to evaluate for relevant studies.

**Study Selection:**

All studies involving the use of RAGT in adults (≥18y) diagnosed with acute inflammatory demyelinating polyneuropathy or GBS were included. Eligible study designs comprised case reports, case series, feasibility studies, and randomized controlled trials. The search yielded 11 potentially relevant articles.

**Data Extraction:**

Study selection was conducted in 2 stages: title and abstract screening followed by full-text review. Discrepancies between the 2 independent reviewers were resolved through discussion until consensus was achieved.

**Data Synthesis:**

Four studies met the inclusion criteria, comprising 2 case reports, 1 mixed-methods feasibility study, and 1 cross-sectional study, with a total of 19 participants. Across studies, RAGT was generally reported to be feasible, well tolerated, and acceptable to patients, with no major safety concerns. Reported benefits included the ability to initiate overground ambulation, high-intensity repetitive gait practice, and reduced physical burden on therapists. Patient acceptance was supported by positive user experiences, adherence to training sessions, and completion of prescribed protocols, although outcome measures and reporting were inconsistent.

**Conclusions:**

The current evidence base for RAGT in adults with GBS is limited but suggests that it is a feasible and acceptable adjunct to conventional rehabilitation. Although functional improvements were reported, conclusions regarding effectiveness remain constrained by small sample sizes and study heterogeneity. Further prospective and methodologically robust studies are needed to better characterize feasibility, patient experience, and implementation considerations of RAGT in this population.

Guillain–Barré syndrome (GBS) is a form of acute inflammatory demyelinating polyneuropathy (AIDP), an autoimmune condition affecting the peripheral nerves. This may result in debilitating neurologic deficits, including weakness, and sensory impairments, usually resulting in their inability to ambulate.^1^ Despite early treatment with intravenous immunoglobulin (IVIG) and plasma exchange therapy, the clinical course varies between each individual, with varying severities. Often, patients would require a prolonged period of inpatient rehabilitation to regain their function.

Robot-assisted gait training (RAGT) in rehabilitation as an adjunct to conventional rehabilitation techniques has been well established in multiple neurologic conditions, including stroke, spinal cord injury, multiple sclerosis, cerebral palsy, and traumatic brain injury.^2^^,^^3^ RAGT systems allow high-intensity, repetitive, and programmable gait training with standardized movement patterns suited to individual capabilities.^4^ The Robot Institute of America defines a robot as a programmable, multifunctional manipulator designed to move material, parts, or specialized devices through variable programmed motions for the performance of a variety of tasks.^5^ RAGT uses advanced robotic devices and systems designed to support, enhance, and guide the lower limbs during walking.^6^ These robotic devices can generally be divided into exoskeletons and end-effector devices. Exoskeletons are wearable robots that are strapped to the lower limbs and have electrically actuated motors that control joint motion to automate overground walking.^7^ One example of an exoskeleton device is the Lokomat.^a,^^8^ End-effector devices are based on the principle that a patient’s feet are placed on footplates, whose trajectories simulate the stance and swing phases during gait training, an example of an end-effector device is the gait trainer.^8^

Despite growing interest in RAGT for neurologic rehabilitation, the feasibility, strengths, and limitations of its use in adults with GBS remain unclear because of the limited and heterogeneous body of available evidence. A comprehensive mapping of the existing literature is therefore needed to examine the feasibility and practical considerations of RAGT in the rehabilitation of gait among adult patients with GBS.

## Methods

### Problem statement

The aim of this scoping review is to review the current literature on the feasibility as well as weaknesses and strengths of robot-assisted overground gait training in the rehabilitation of gait in adult patients with the GBS.

### Search strategy

This scoping review was reported using the Preferred Reporting Items for Systematic Reviews and Meta-analysis for Scoping Reviews checklist. This review was registered with Open Science Framework (Registration DOI - 10.17605/OSF.IO/NFPRK). A comprehensive search was performed using 3 databases (PubMed, Cochrane, and Embase) CADTH Grey Matters and citation mining of existing search results from November 1, 2024 to May 2, 2025. Searches were performed monthly to ascertain if there were new literature over the course of 6 months. The keywords in the search strategies include “acute demyelinating polyneuropathy,” “Guillain barré syndrome,” “Rehabilitation,” and “Robotics.”

On the basis of the results generated and the review of relevant articles, the citations were reviewed and the relevant articles from the citations were included in this review.

### Eligibility criteria

#### Inclusion criteria

All studies including and not limited to case reports, case series, feasibility studies, observational studies, and randomized controlled trials involving adults (≥18y) diagnosed with AIDP or GBS that used RAGT as part of rehabilitation were included.

#### Exclusion criteria

Studies were excluded if they did not specifically involve patients with AIDP or GBS, if robotic therapy was not used for gait training or ambulation, or if the study population consisted exclusively of pediatric patients (<18y).

### Study screening, data abstraction, and selection

By applying the eligibility criteria, 2 reviewers with a rehabilitation medicine background (a senior consultant (S.K.L. – 15y experience) in rehabilitation medicine, and a senior resident (J.s.S. – 2y experience in rehabilitation medicine) screened the articles for selection. The first selection was from the title and abstract screening, and the second was from the full-text screening. All conflicts generated during the screening stages between the 2 reviewers were discussed until a consensus was reached.

## Results

**Fig 1:**
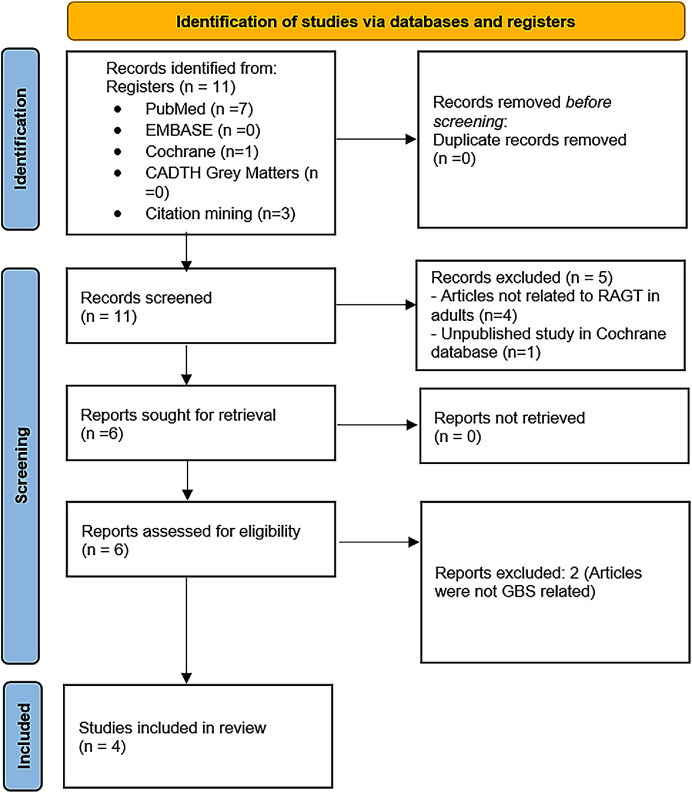
Identification of studies via database and registries.

On the basis of the predefined search strategy, a total of 11 articles were identified, including 7 from PubMed, 1 from the Cochrane Library, none from Embase, and 3 through citation mining. After title and abstract screening, 4 articles met the inclusion criteria and were retained for full-text review. Of these, 2 were case reports, 1 was a mixed-methods feasibility study, and 1 was a cross-sectional study (fig 1). Across the included studies, a total of 19 adult patients were reported. The results are further summarized in table 1.

**Table 1:** Summary of results.

Authors	Publication (Y)	Type of Study	No. of Patients	Patient Description	Type of RAGT	Regime and Duration of Rehabilitation	Adverse Outcomes
Yabuki et al^9^	2024	Case report	1	78-year-old man, with GBS in the chronic phase	Hip-wearable exoskeleton Robot (HWA-01) (exoskeleton)	16 wk of rehabilitation, with 3-4 weekly alternation between conventional gait training and robotic assisted rehabilitation with the hip-wearable exoskeleton robot	None
Chen et al^10^	2024	Case report	1	75-year-old woman, with GBS in the acute phase	Lokomat (Exoskeleton)	2 sessions of RAGT on third week and fourth week of admission	None
Hotz et al^11^	2024	Mixed method feasibility study	2	1 patient in the acute phase and 1 in the chronic phase of GBS	LEXO1 robotic gait trainer, Tyromotion (end-effector)	RAGT for 45 min, 4 times/wk, for 4 wk	None
Rhee et al^12^	2020	Cross-sectional study	15	15 patients with mean age of 55.7 y with GBS (phase not included in article)	Morning Walk (End-effector)	24 sessions of 30-min sessions	1 minor adverse outcome of pain related to the saddle while using the device

Yabuki et al^9^ described the use of a hip-wearable exoskeleton in a 78-year-old man who was diagnosed with GBS. The patient was transferred to inpatient rehabilitation at day 185 of disease onset. The patient initially underwent conventional rehabilitation, including physiotherapy and occupational therapy for 7 months. He was already ambulating at supervision to independent level by the time he underwent robot-assisted rehabilitation. On day 382 from disease onset, he started on the exoskeleton. The aim of this robotic device was to improve his gait and to achieve independent ambulation. The primary outcomes were comfortable walking speed, stride length, and cadence, and secondary outcomes were GBS disability score, Medical Research Council (MRC) sum, 6-minute walk test (6MWT), motor items of Functional Independence Measure (FIM) (motor-FIM), and Overall Neuropathy Limitations Scale scores. The patient underwent 16 weeks of rehabilitation, with 3-4 weekly alternating between conventional gait training and robot-assisted rehabilitation with the hip-wearable exoskeleton robot. The authors concluded that after completion of a 16-week combined conventional rehabilitation and RAGT, there were improvements in gait ability, MRC score, 6MWT, Overall Neuropathy Limitations Scale, and motor-FIM.

In a case study published by Chen et al,^10^ the use of a RAGT device Lokomat^a^ (a type of robotic exoskeleton) in a patient diagnosed with GBS was reported. She received a 5-day course of IVIG. Despite intact strength in her upper limbs, she had decreased muscle strength (MRC grade 0/5) in her lower limbs. She received conventional therapy, which included physical and occupational therapy to improve her lower limb muscle strength, sitting balance, transfer technique, standing balance, ambulation, and gait balance 5 days a week, with a gradual increase in duration to a total of 1 hour, and was adjusted accordingly based on her physical condition during each session. In addition, the patient underwent 2 sessions of RAGT on the 3rd (24-min session) and 4th (30-min session) weeks of admission. One month after hospital admission, she improved from being bedridden to regaining the ability to sit and was able to transfer out of bed to upright standing with walker support. The MRC grade of her lower limbs increased to 4/5. Her Barthel Index score for activities of daily living improved from 15 to 65. However, she still required moderate assistance for short-distance ambulation.

In a mixed-methods feasibility study published by Hotz et al,^11^ the feasibility of LEXO1 robotic gait trainer (Tyromotion),^b^ a type of end-effector, under various neurologic conditions was studied. Twenty-six patients with various neurologic diseases were recruited to receive RAGT as part of the rehabilitation program, including 2 patients with acute/chronic inflammatory polyneuropathy. Patients received RAGT for 45 minutes, 4 times per week, for 4 weeks. The secondary outcomes included the 10-meter walk test, 6MWT, timed Up and Go, and Functional Gait Assessment. Both patients who underwent the RAGT showed improvements in the 10MWT, 6MWT, timed Up and Go, and Functional Gait Assessment. No adverse events were reported in this study.

From citation mining of the reviewed articles, an additional article was identified, Rhee et al,^12^ published in 2020, which was a cross-sectional study, focusing on effects of a type of an end-effector robotic device with provision of body support, via the use of a saddle seat, for rehabilitation on patients with GBS. A total of 15 patients underwent RAGT (Morning Walk device)^c^ rehabilitation consisting of twenty-four 30-minute sessions. The primary outcome measure was to evaluate the effectiveness of RAGT in patients with GBS using an end-effector type robotic device. The outcome measures collected were the MRC scale, Functional Ambulation Categories for measuring functional gait; Modified Barthel Index; Rivermead Mobility Index for testing functional abilities; and 2-minute walk test for measuring endurance of walking distance. The study reported that after RAGT, there were improvements in the MRC score of the hip, knee, and ankle, Functional Ambulation Categories, Modified Barthel Index, 2-minute walk test, and Rivermead Mobility Index suggesting improvement in strength, gait function, endurance, function, and activities of daily living.

## Discussion

GBS is characterized by acute neuromuscular paralysis with associated sensory and motor deficits that typically progress from distal to proximal muscle groups. Acute management focuses on immunomodulatory therapies such as IVIG and/or plasma exchange therapy to stabilize disease progression and improve prognosis. Despite timely medical treatment, approximately 40%-67% of patients with GBS require inpatient rehabilitation.^13^ Persistent impairments commonly include respiratory muscle weakness, autonomic dysfunction, pain, fatigue, immobility, and dysphagia, often resulting in a prolonged period of functional limitation. This extended recovery phase highlights the need for rehabilitation strategies that support early, safe, and progressive mobilization, making interventions such as RAGT particularly relevant from a feasibility perspective.

The primary goal of rehabilitation in GBS is to prevent secondary complications and facilitate the recovery of functional independence once patients are medically stable, involving a multidisciplinary team consisting of a physiotherapist, occupational therapist, speech therapist, social worker, and nurse, led by a rehabilitation medicine physician.^14^ Rehabilitation is typically delivered by a multidisciplinary team and is guided by principles of early mobilization, prevention of complications related to immobility, and the gradual progression of exercise intensity while avoiding excessive fatigue.^15^^,^^16^ Within this framework, RAGT may be a feasible adjunct to conventional therapy by enabling controlled, repetitive, and task-specific gait practice while adhering to established rehabilitation principles for GBS.

Although there is currently no consensus regarding the routine use of robotic devices for ambulation in patients with GBS, the limited available literature suggests that RAGT is feasible and generally well tolerated in this population. The studies included in this scoping review demonstrated considerable heterogeneity in terms of type of robot, rehabilitation protocols, duration of training, and timing of intervention across different disease phases. Despite this variability, a consistent finding across studies was that patients were able to participate in RAGT sessions and complete prescribed training protocols, indicating acceptable levels of tolerance and adherence. The systematic review by Martino Cinnera et al^17^ also showed that there is potential in improvement of motor function of lower extremity without adverse events. These findings support the feasibility of incorporating RAGT into rehabilitation programs for selected patients with GBS. This is further supported with studies that have shown that RAGT can potentially reduce the physical burden on physiotherapists while ensuring safety during physical mobilization.^18^^,^^19^

Patient acceptance of RAGT appears to be influenced by its potential to initiate ambulation earlier in the recovery process, particularly in those who have not yet regained sufficient strength or endurance for conventional overground walking. The assistive capabilities of robotic devices allow patients to practice gait cycles with increased repetition and intensity while maintaining safety, which may enhance motivation and engagement in therapy. Although the precise mechanisms underlying functional improvement in GBS remain unclear, we postulate that RAGT facilitates task-specific motor retraining by enabling symmetrical and coordinated lower limb movements. For example, Yabuki et al^9^ reported that hip-wearable exoskeletons provide phase-specific assistance during stance and swing, promoting coordinated bilateral movement and supporting early gait practice.

Advances in RAGT technology, including adjustable degrees of freedom and assist-as-needed control strategies, further enhance feasibility by allowing individualized training parameters based on patient capability and tolerance. These features enable high-repetition, high-intensity gait practice while mimicking physiological movement patterns, which may be particularly advantageous for patients with fluctuating strength and endurance, as commonly observed in GBS.^10^

Importantly, similar technologies have demonstrated high levels of feasibility and positive patient experiences in other neurologic populations, including stroke, spinal cord injury, and multiple sclerosis, where studies have reported physical, psychological, and social benefits.^18^^,^^20^ In the stroke population, studies have demonstrated that poststroke patients with initial reduced ambulatory ability were able to walk longer distances during physiotherapy sessions with the use of robot-assisted exoskeleton, this in turn enables restoration of muscle coordination in the affected limb with improvements of gait patterns.^21^^,^^22^ In spinal cord injury population, robotic exoskeletons have been shown to improve walking balance, lower extremity strength, improvements in bowel and bladder function, bone density, spasticity, pain and cardiovascular function, functional scores, and respiratory function compared with conventional gait training.^23^, ^24^, ^25^, ^26^, ^27^ Nonphysical benefits of RAGT have also been reported in this population, where patients reported psychological improvements and enhanced sense of community integration.^28^^,^^29^ Robotics in lower extremity rehabilitation have also been shown to induce convergent neurophysiological signatures in several neurologic conditions, where end-effector devices tend to re-engage ipsilesional motor cortex, whereas overground exoskeletons tend to improve network-level efficiency.^30^ Although these findings cannot be directly extrapolated to GBS, they provide supportive context for the acceptability of RAGT in neurologic rehabilitation more broadly.

Safety and tolerability are critical components of feasibility. Across the studies included in this scoping review, only 1 adverse event was reported, involving pain and discomfort related to the device interface.^12^ This aligns with broader evidence from stroke and spinal cord injury populations, where RAGT has generally been shown to be safe and well tolerated.^31^, ^32^, ^33^ However, sensory disturbances commonly present in GBS, such as neuropathic pain, paresthesia, and allodynia, pose a potential challenge in using RAGT in this group of patients.^1^ Robotic devices are rigid and require good skin contact to provide the support required for overground ambulation that may in turn pose a challenge when donning the device, as it may be painful for patients and they may be unable to tolerate the use of robotic devices in rehabilitation. Another potential challenge is that patients with GBS frequently experience orthostatic hypotension because of autonomic nervous system dysfunction, necessitating close monitoring during sitting or standing to prevent hypotension-related complications.^1^ This may limit the tolerability of RAGT in this group of patients because of significant blood pressure fluctuations during ambulation. Therefore, careful patient selection, gradual familiarization, and attention to comfort during donning and training while monitoring the postural blood pressure are essential to optimize tolerability.

Finally, the high cost of robotic devices remains a significant barrier to widespread implementation and represents an important feasibility consideration at the systems level.^34^ Capital costs, maintenance, and training requirements may limit accessibility in many rehabilitation settings. For instance, the price of Lokomat^a^ was €881,292 in 2022, whereas the price of an overground robotic exoskeleton was USD 150,000, excluding maintenance and repair costs.^35^^,^^36^ Although preliminary economic analyses suggest that robot-assisted therapy may be cost-effective in specific populations, such as individuals with complete spinal cord injury, further research is needed to evaluate cost-effectiveness in GBS rehabilitation.^34^

In summary, although evidence remains limited, this scoping review suggests that RAGT is a feasible and generally acceptable adjunct to rehabilitation for adults with GBS. Current findings support its tolerability, safety, and practical implementation in selected patients, although heterogeneity in study design and intervention protocols precludes conclusions regarding effectiveness. A recent study has proposed a protocol to assess the effectiveness of robotic devices for motor recovery in GBS, but this remains preliminary and has not yet progressed beyond the protocol stage.^17^ Future research involving RAGT in people with GBS should prioritize feasibility outcomes, patient-reported experiences, standardized reporting of adverse events, implementation considerations as well as addition of neurophysiological and neuroimaging techniques for evaluation of neuroplasticity, to better inform clinical decision-making in this population.

### Study limitations

At the time of this scoping review, the available literature on RAGT in patients with GBS is sparse and primarily consists of small observational studies and case reports. As a result, the overall body of evidence is limited, precluding definitive conclusions regarding effectiveness and restricting the generalizability of findings related to feasibility and patient acceptance.

In addition, substantial heterogeneity was observed across included studies with respect to type of robotic device, training protocols, session frequency and duration, and timing of intervention during the disease course. This variability limits direct comparison between studies and precludes head-to-head evaluation of different robotic devices or training approaches. Nevertheless, despite these differences, the underlying principles of RAGT application in GBS rehabilitation were broadly similar across studies.

Furthermore, none of the included studies directly compared RAGT with conventional gait rehabilitation in a controlled manner. Consequently, it is not possible to determine whether observed functional improvements or patient-reported benefits can be attributed specifically to RAGT rather than to concurrent therapies or the natural recovery trajectory of GBS, which is known to vary widely depending on disease severity and individual patient factors.

## Conclusions

The use RAGT in neurorehabilitation continues to expand across a variety of neurologic conditions, including GBS. Although the current evidence base for RAGT in GBS is limited, available studies suggest that these devices are feasible, generally well tolerated, and acceptable to patients, particularly during the early phases of rehabilitation when motor control, balance, and endurance are impaired. RAGT may facilitate early, task-specific, and high-repetition gait training while supporting safety and reducing the physical burden on therapists, making it a promising adjunct to conventional rehabilitation in selected patients.

Challenges to implementation include sensory disturbances, orthostatic hypotension, and the need for careful patient selection, gradual familiarization, and close monitoring during training. High costs and limited availability of robotic devices also remain important barriers to widespread adoption. Nevertheless, advances in technology, including adjustable and assist-as-needed robotic devices, and the potential for reduced costs with evolving manufacturing techniques, may increase accessibility and practicality in the future.

Given the current small number of studies, heterogeneity in devices and protocols, and absence of controlled comparisons with conventional rehabilitation, definitive conclusions regarding effectiveness of RAGT cannot be drawn. Future research should prioritize systematically evaluating feasibility, patient-reported outcomes, safety, and implementation considerations. Standardized reporting of adverse events and protocols will be essential to inform clinical decision-making and optimize the use of RAGT in adults with GBS.

## Suppliers

a. Lokomat. (Hocoma)

b. LEXO1 robotic gait trainer. (Tyromotion)

c. Morning Walk device. (CUREXO Inc)

## Disclosure

The investigators have no financial or nonfinancial disclosures to make in relation to this project.

## Authorship contributions/CRediT statements

J.S.S. contributed to the study design, analysis of results, and writing of the manuscript. S.K.L. conceived the idea for the project, supervised the project, and writing of the manuscript.

## Data statements

All data generated or analyzed during this study are included in this published article.

## References

[1] Leonhard S.E., Mandarakas M.R., Gondim F.A.A. (2019). Diagnosis and management of Guillain–Barré syndrome in ten steps. Nat Rev Neurol.

[2] Hidler J., Sainburg R. (2011). Role of robotics in neurorehabilitation. Top Spinal Cord Inj Rehabil.

[3] Koda M., Kubota S., Kadone H. (2023). Robotic rehabilitation therapy using Hybrid Assistive Limb (HAL) for patients with spinal cord lesions: a narrative review. N Am Spine Soc J.

[4] Lee J.H., Kim G. (2025). Effectiveness of robot-assisted gait training in stroke rehabilitation: a systematic review and meta-analysis. J Clin Med.

[5] Morone G., Paolucci S., Cherubini A. (2017). Robot-assisted gait training for stroke patients: current state of the art and perspectives of robotics. Neuropsychiatr Dis Treatment.

[6] Park Y.H., Lee D.H., Lee JH. (2024). A comprehensive review: robot-assisted treatments for gait rehabilitation in stroke patients. Medicina (Kaunas).

[7] Louie D.R., Eng JJ. (2016). Powered robotic exoskeletons in post-stroke rehabilitation of gait: a scoping review. J Neuroeng Rehabil.

[8] Mehrholz J., Kugler J., Pohl M., Elsner B. (2025). Electromechanical-assisted training for walking after stroke. Cochrane Database Syst Rev.

[9] Yabuki J., Yoshikawa K., Koseki K., Ishibashi K., Matsushita A., Kohno Y. (2024). Improvement of functional mobility using a hip-wearable exoskeleton robot in Guillain-Barré syndrome with residual gait disturbance: a case report. Cureus.

[10] Chen F.Y., Hou W.H., Lee H.H., Huang Y.C., Siow CY. (2024). Additional rehabilitative robot-assisted gait training for ambulation in geriatric individuals with Guillain-Barré syndrome: a case report. Medicina (Kaunas).

[11] Hotz I., Mildner S., Stampfer-Kountchev M. (2024). Robot-assisted gait training in patients with various neurological diseases: a mixed methods feasibility study. PLoS One.

[12] Rhee SY, Jeon H, Kim SW, Lee JS. The effect of an end-effector type of robot-assisted gait training on patients with Guillain-Barre syndrome: a cross-sectional study. 2020;F1000Research:9:1465.

[13] Foster E., Bonavia L., Subramaniam A., Green C., Butler E., Tiruvoipati R. (2016). A descriptive study of patients with Guillain-Barré syndrome: experience from an Australian tertiary level hospital. AMJ.

[14] Van den Bergh P.Y.K., van Doorn P.A., Hadden R.D.M. (2021). European Academy of Neurology/Peripheral Nerve Society guideline on diagnosis and treatment of chronic inflammatory demyelinating polyradiculoneuropathy: report of a joint task force-second revision. Eur J Neurol.

[15] Meythaler J M (1997). Rehabilitation of Guillain-Barré syndrome. Arch Phys Med Rehabil.

[16] Doets A.Y., Lingsma H.F., Walgaard C. (2022). Predicting outcome in Guillain-Barré syndrome: international validation of the Modified Erasmus GBS Outcome Score. Neurology.

[17] Martino Cinnera A., D'Arienzo M., Piatti D. (2024). Robot-Assisted Therapy in Guillain-Barrè syndrome: systematic review of primary evidence and study protocol for a randomized clinical trial. J Clin Med.

[18] Yang J., Zhu Y., Li H., Wang K., Li D., Qi Q. (2024). Effect of robotic exoskeleton training on lower limb function, activity and participation in stroke patients: a systematic review and meta-analysis of randomized controlled trials. Front Neurol.

[19] Banyai A.D., Brișan C. (2024). Robotics in physical rehabilitation: systematic review. Healthcare (Basel).

[20] Cumplido-Trasmonte C., Molina-Rueda F., Puyuelo-Quintana G. (2023). Satisfaction analysis of overground gait exoskeletons in people with neurological pathology. a systematic review. J Neuroeng Rehabil.

[21] Tam P.K., Tang N., Kamsani N.S.B. (2025). Overground robotic exoskeleton vs conventional therapy in inpatient stroke rehabilitation: results from a pragmatic, multicentre implementation programme. J Neuroeng Rehabil.

[22] Lee M-H, Tian M-Y, Kim M-K. (2024). The effectiveness of overground robot exoskeleton gait training on gait outcomes, balance, and motor function in patients with stroke: a systematic review and meta-analysis of randomized controlled trials. Brain Sci.

[23] Liu S., Chen F., Yin J., Wang G., Yang L. (2025). Comparative efficacy of robotic exoskeleton and conventional gait training in patients with spinal cord injury: a meta-analysis of randomized controlled trials. J Neuroeng Rehabil.

[24] Esquenazi A., Talaty M., Packel A., Saulino M. (2012). The ReWalk powered exoskeleton to restore ambulatory function to individuals with thoracic-level motor-complete spinal cord injury. Am J Phys Med Rehabil.

[25] Chun A., Asselin P.K., Knezevic S. (2020). Changes in bowel function following exoskeletal-assisted walking in persons with spinal cord injury: an observational pilot study. Spinal Cord.

[26] Karelis A., Carvalho L., Castillo M., Gagnon D., Aubertin-Leheudre M. (2017). Effect on body composition and bone mineral density of walking with a robotic exoskeleton in adults with chronic spinal cord injury. J Rehabil Med.

[27] Mekki M., Delgado A.D., Fry A., Putrino D., Huang V. (2018). Robotic rehabilitation and spinal cord injury: a narrative review. Neurotherapeutics.

[28] Cahill A., Ginley O.M., Bertrand C., Lennon O. (2018). Gym-based exoskeleton walking: a preliminary exploration of non-ambulatory end-user perspectives. Disabil Health J.

[29] Charbonneau R., Loyola-Sanchez A., McIntosh K., MacKean G., Ho C. (2022). Exoskeleton use in acute rehabilitation post spinal cord injury: a qualitative study exploring patients’ experiences. J Spinal Cord Med.

[30] Calabrò R.S., Calderone A., Simoncini L. (2026). The potential of robotics: a systematic review of neuroplastic changes following advanced lower limb rehabilitation in neurological disorders. Neurosci Biobehav Rev.

[31] Gandolfi M., Geroin C., Tomelleri C. (2017). Feasibility and safety of early lower limb robot-assisted training in sub-acute stroke patients: a pilot study. Eur J Phys Rehabil Med.

[32] Lin Y.N., Huang S.W., Kuan Y.C. (2022). Hybrid robot-assisted gait training for motor function in subacute stroke: a single-blind randomized controlled trial. J Neuroeng Rehabil.

[33] Park J.M., Kim Y.W., Lee S.J., Shin JC. (2024). Robot-assisted gait training in individuals with spinal cord injury: a systematic review and meta-analysis of randomized controlled trials. Ann Rehabil Med.

[34] Pinto D., Garnier M., Barbas J. (2020). Budget impact analysis of robotic exoskeleton use for locomotor training following spinal cord injury in four SCI Model Systems. J Neuroeng Rehabil.

[35] Klobucká S., Klobucký R., Valovičová K., Šiarnik P., Kollár B. (2023). Cost-effectiveness analysis of robot-assisted gait training in patients with bilateral spastic cerebral palsy. Cost Eff Resour Alloc.

[36] Pinto D., Heinemann A.W., Chang S.H. (2023). Cost-effectiveness analysis of overground robotic training versus conventional locomotor training in people with spinal cord injury. J Neuroeng Rehabil.

